# LigaSure small jaw versus conventional neck dissection: a systematic review and meta-analysis

**DOI:** 10.1186/s40463-021-00504-2

**Published:** 2021-03-29

**Authors:** Tai-Yu Chen, Li-Jen Hsin, Wan-Ni Lin, Ming-Shao Tsai, Yao-Te Tsai, Yi-Chan Lee

**Affiliations:** 1 Department of Otolaryngology - Head and Neck Surgery, Chang Gung Memorial Hospital, Taoyuan, Taiwan; 2 College of Medicine, Chang Gung University, Taoyuan, Taiwan; 3 Department of Otolaryngology—Head and Neck Surgery, Chang Gung Memorial Hospital, Chiayi, Taiwan; 4 Department of Otolaryngology - Head and Neck Surgery, Chang Gung Memorial Hospital, No.222, Maijin Rd., Anle Dist, Keelung City, 204 Taiwan

**Keywords:** LigaSure vessel sealing system, LigaSure small jaw, Electrothermal bipolar vessel sealing, Neck dissection, Neck lymphadenectomy

## Abstract

**Background:**

Neck dissection has a central role in the management of head and neck cancers. This systematic review aimed to compare the intraoperative and postoperative parameters between conventional and LigaSure Small Jaw (LSJ)-assisted neck dissection.

**Methods:**

PubMed (MEDLINE), Embase, and the Cochrane Library were searched.

independently by two authors for relevant articles comparing the outcomes of conventional and LSJ-assisted neck dissection. Data from each study were extracted, and a random-effects model was used in the pooled analysis.

**Results:**

Compared with conventional techniques, LSJ-assisted neck dissection was associated with a significantly reduced operative time. The rates of postoperative hematoma, infection, amount of intraoperative blood loss, the length of hospital stay and the drainage amount showed no significant intergroup differences.

**Conclusions:**

The meta-analysis provides evidence that properly using LSJ may reduce the operative time compared with that of conventional techniques. Surgeons may consider using LSJ in neck dissection according to personal experiences.

**Graphical abstract:**

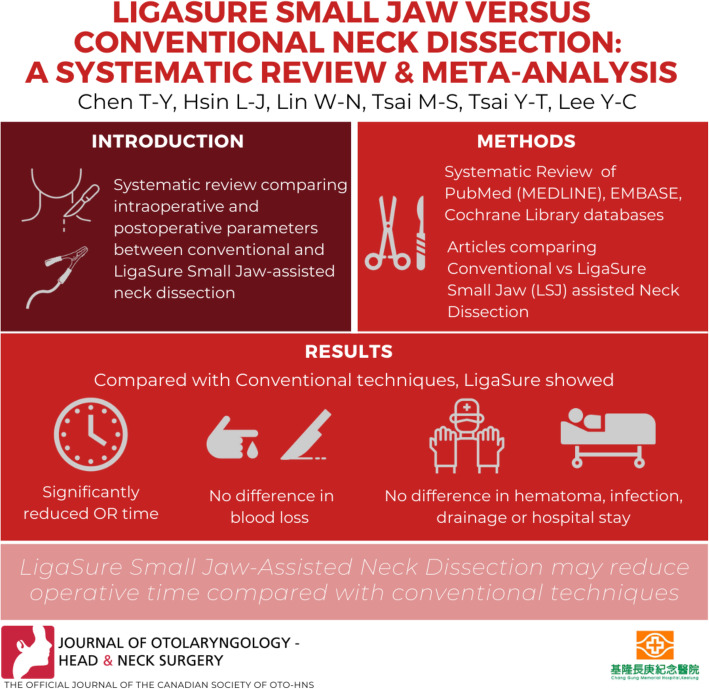

**Supplementary Information:**

The online version contains supplementary material available at 10.1186/s40463-021-00504-2.

## Background

The standard form of neck dissection (ND) was proposed by Crile for the first time in 1906 [1]. Several modifications, such as modified radical ND, selective ND and extended ND, have been subsequently developed and employed as a central procedure in the management of head and neck cancer [1]. Careful dissection and ligation of vessels are both critical procedures in ND. Conventionally, these steps have been achieved with suture ligation, hemoclips, and electrocoagulation [2–6]. Several energy-based devices have been introduced in recent years to facilitate ligation procedures and decrease lateral heat dispersion [7–10]. LigaSure Small Jaw (LSJ), an energy-based device, is a bipolar vessel-sealing instrument that also incorporates a tissue divider. The feasibility and safety of LSJ have been reported in several surgeries, such as thyroidectomy, hemorrhoidectomy and mastectomy [11–13]. The aim of the present study was to compare intraoperative and postoperative parameters between LSJ and conventional ND in the existing English literature.

## Methods

### Data collection and data sources

The present study was conducted according to the Preferred Reporting Items for Systematic Reviews and Meta-Analyses (PRISMA) statement. Two of the authors (TYC and YCL) searched PubMed, Embase, and the Cochrane Library independently and extensively for articles of interest published before June 2020. The keywords used in the search process included “LigaSure vessel sealing system”, “LigaSure Small Jaw”, “neck dissection”, “neck lymphadenectomy”, “cervical lymphadenectomy” and “lymph node dissection”. Moreover, these two authors reviewed the reference lists of the included studies to identify additional articles.

### Study selection and data extraction

The inclusion criteria were studies including patients with head and neck cancers who underwent ND, articles published in English, and studies comparing the outcomes of ND between the LSJ and conventional techniques. The exclusion criteria were based primarily on the absence of one of the inclusion criteria. Studies without a control group, studies using the same database, articles not published in English, duplicate studies, case reports, abstracts, letters to the editor, and articles pending publication of the full text were excluded from the present study. Data were independently extracted by 2 researchers (TYC and YCL). The bias of the included articles was assessed independently by the two researchers (TYC and YCL) using the Newcastle-Ottawa Scale and the Cochrane Collaboration’s risk of bias tool (RoB 1.0) for nonrandomized and randomized studies, respectively [14, 15]. Discrepancies in study bias assessment were discussed between the two authors until consensus was achieved.

### Outcomes

The main outcomes of this study included operative time, intraoperative blood loss, incidences of postoperative hematoma, incidences of postoperative surgical site infection and length of hospital stay.

### Data analysis

The results were analyzed using Comprehensive Meta-Analysis software, version 3 (Biostat). Standardized mean differences (SMDs) and mean differences (MDs) were calculated to compare the total operative time, the amount of intraoperative blood loss and the length of hospital stay between the LSJ-assisted ND and conventional ND groups. Risk differences (RDs) were calculated to compare the incidences of postoperative hematoma and surgical site infection between the two groups. The overall effect was pooled using a random-effects model. The types of neck dissection in conventional and LSJ-assisted ND group were compared using the using the chi-square test. Statistical heterogeneity among studies was measured using the *I*^*2*^ statistic, which calculated the proportion of overall variation attributable to between-study heterogeneity. An *I*^*2*^ statistic exceeding 50% indicates moderate heterogeneity, and an *I*^*2*^ statistic exceeding 75% indicates high heterogeneity [16]. Potential publication bias was assessed using a funnel plot and the Egger’s intercept test [16]. Any 2-sided *P-*value less than 0.05 was considered statistically significant.

## Results

### Study selection

**Fig. 1:**
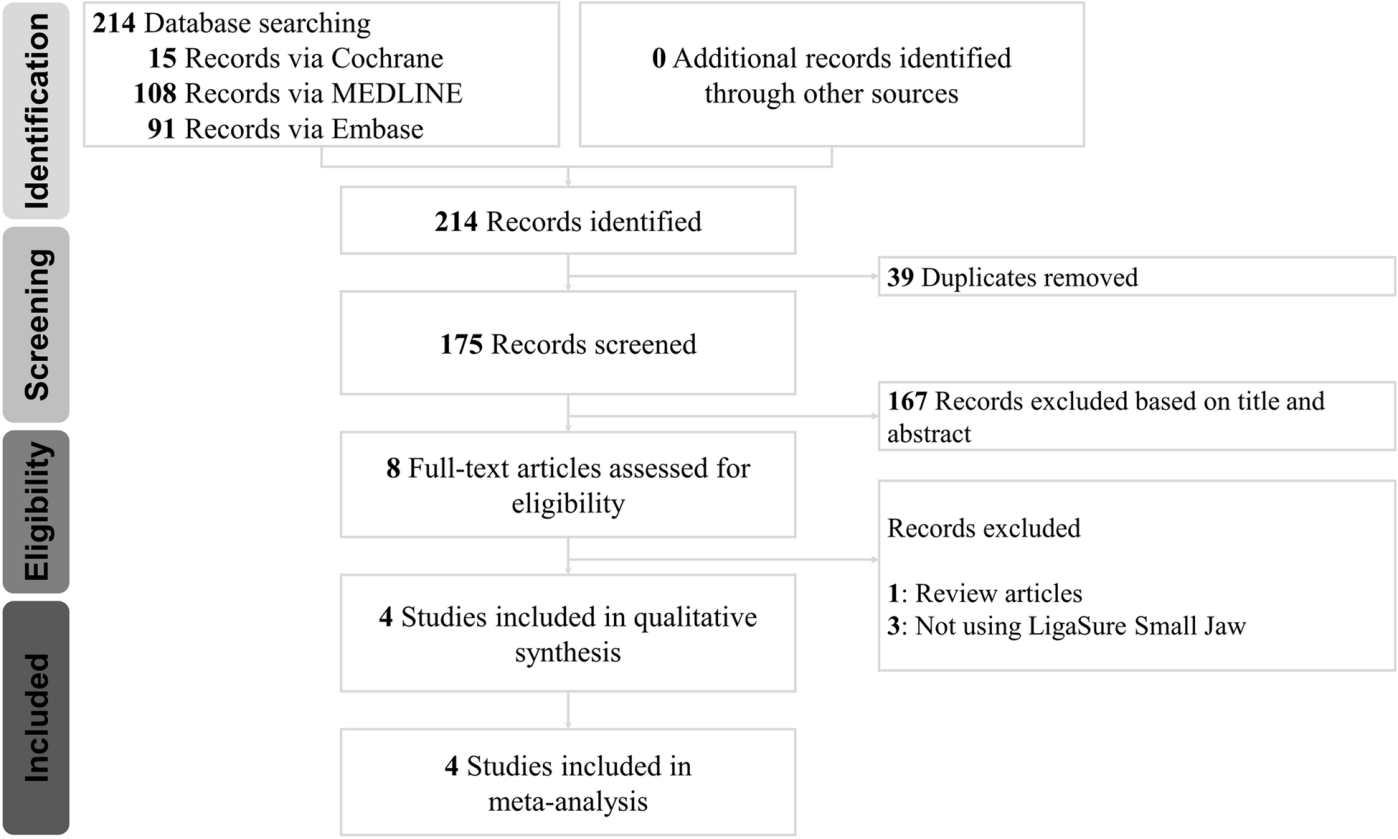
Flow Diagram of the Literature Search

The initial literature search yielded a total of 214 articles, and 39 duplicates were removed. A total of 167 articles were also excluded based on their titles and abstracts. The remaining 8 potentially eligible studies were retrieved for a careful review of the full texts. Among them, review articles and studies not using LSJ were excluded from the meta-analysis. Finally, 4 articles were included in this review [2–4, 6]. A flow diagram describing the process involved in study identification and inclusion/exclusion is shown in Fig. 1. eTable 1 in the Supplement summarizes the literature search process and the keywords used.

### Demographics

Table 1 lists the basic demographics of patients from the 4 articles, including 1 randomized study and 3 nonrandomized studies. The pooled prevalence of comprehensive ND was comparable between the two groups (*P* = 0.75). The bias assessment for each study is described in eTable 2 and eTable 3 in the Supplement.

**Table 1:** Basic characteristics of the included studies

Authors	Year	Country	Study Design	Mean Age (y)	Sex (M/F)	Type of ND	Number of CNDs		Sample Size^*^	
							LSJ	CT	LSJ	CT
Ozturk et al.	2016	Turkey	Prospective study	62.7	17/8	SND	0	0	15	10
Lin et al.	2017	Taiwan	RCT	52.4	34/7	SND	0	0	21	20
Tirelli et al.	2017	Italy	Retrospective study	66.3	48/20	SND/CND	20	18	32	36
Suzuki et al.	2018	Japan	Retrospective study	67.5	52/14	SND/CND	3	3	30	36
							*23/21*		98	102
							*P =* 0.75^a^			

*LSJ* LigaSure Small Jaw; *CT* Conventional technique; *y* Year; *ND* Neck dissection; *RCT* Randomized controlled trial; *SND* Selective neck dissection;

*CND* Comprehensive neck dissection; *M* Male; *F* Female

^a^ *P*-value from Chi-squared test of the pooled prevalence of CND between the LSJ and CT groups

^*^ Number of sides of neck dissection

### Outcomes

#### Operative time

Four of the included studies reported the operative time required for ND [2–4, 6].

**Fig. 2:**
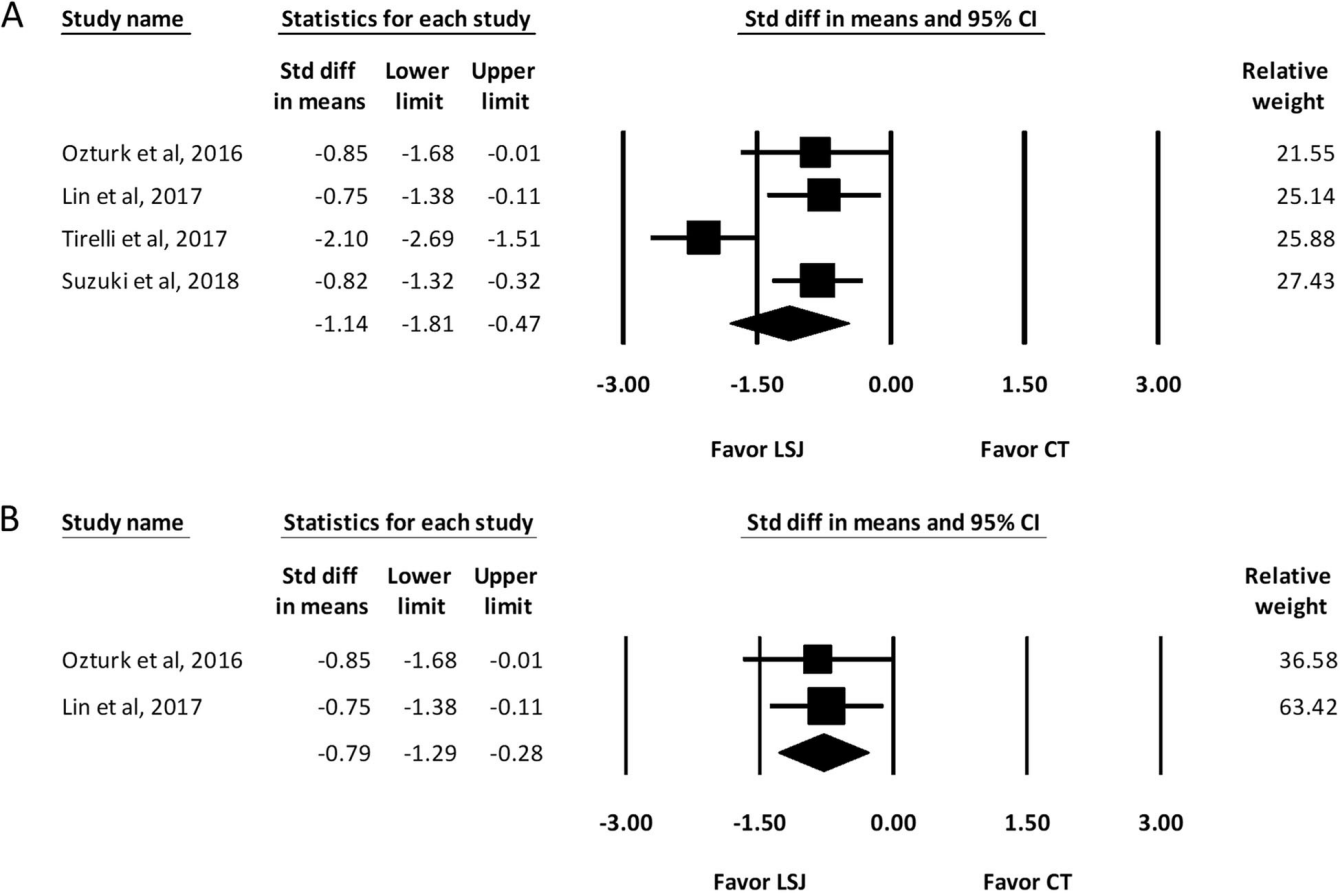
Forest Plot of the Operative Time During Neck Dissection. **a** Overall study group. **b** Studies including only selective neck dissection. Std diff in means, Standardized difference in means; CI, confidence interval; LSJ, LigaSure Small Jaw; CT, conventional technique

Among the four studies included, two types of ND were performed. Comprehensive neck dissection (CND) involved surgical removal of all the five lateral cervical lymph node levels (I-V) and selective neck dissection (SND) involved the removal of less than five levels of lymph nodes. The pooled results of overall study groups showed that the operative time was lower in the LSJ group (SMD, − 1.14; 95% confidence interval [CI], − 1.81 to − 0.47). A reduction in operative time by 29.0 min was observed **(**Fig. 2a**)**. Subgroup analysis of the two studies including only SND also showed that the operative time was lower in the LSJ group (SMD, − 0.79; 95% CI, − 1.29 to − 0.28) [2, 3] (Fig. 2b).

#### Intraoperative blood loss

**Fig. 3:**
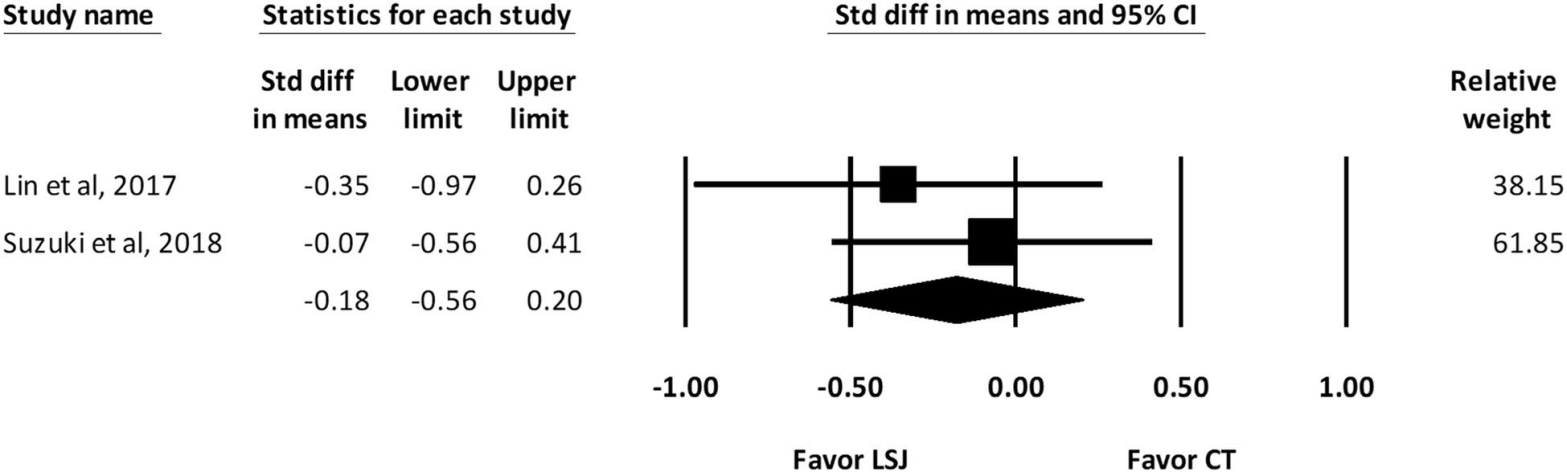
Forest Plot of Intraoperative Blood Loss During Neck Dissection. Std diff in means, Standardized difference in means; CI, confidence interval; LSJ, LigaSure Small Jaw; CT, conventional technique

Two of the included studies recorded the amount of intraoperative blood loss [2, 4]. The pooled analysis showed no significant difference between the LSJ and conventional technique groups regarding intraoperative blood loss (SMD, − 0.18; 95% CI, − 0.56 to 0.20) (Fig. 3).

#### Postoperative hematoma

**Fig. 4:**
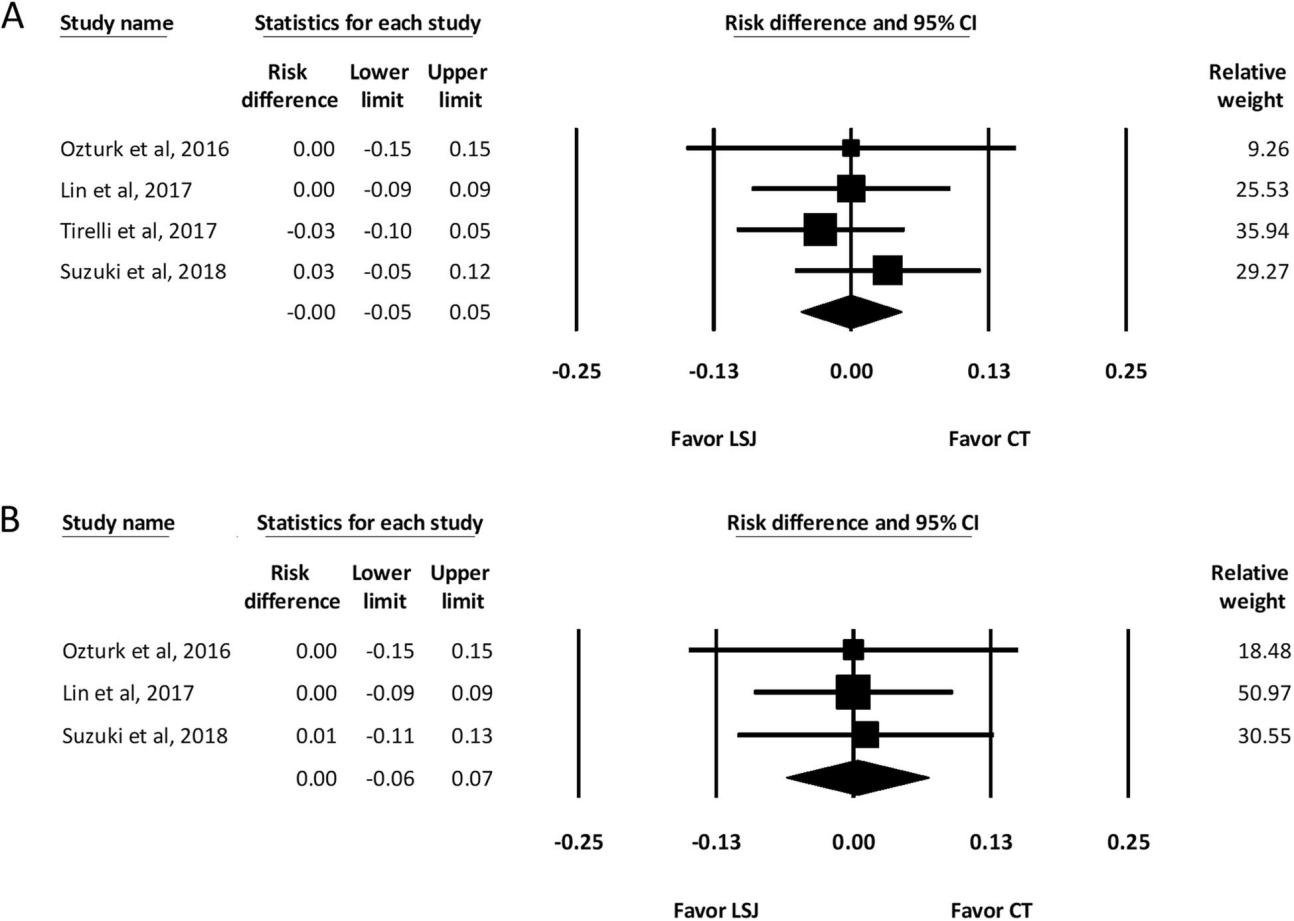
Forest Plot of Postoperative Hematoma and Surgical Site Infection After Neck Dissection. **a** Postoperative hematoma. **b** Postoperative surgical site infection. CI, confidence interval; LSJ, LigaSure Small Jaw; CT, conventional technique

Four of the included studies reported the incidence of postoperative hematoma in both groups [2–4, 6]. The pooled analysis showed no significant difference between the LSJ and conventional technique groups regarding postoperative hematoma (RD, − 0.00; 95% CI, − 0.05 to 0.05) (Fig. 4a).

#### Postoperative surgical site infection

Three of the included studies reported the incidence of surgical site infection [3, 4]. The pooled analysis showed no significant difference between the LSJ and conventional technique groups regarding postoperative surgical site infection (RD, 0.00; 95% CI, − 0.06 to 0.07) (Fig. 4b).

#### Length of hospital stay

**Fig. 5:**
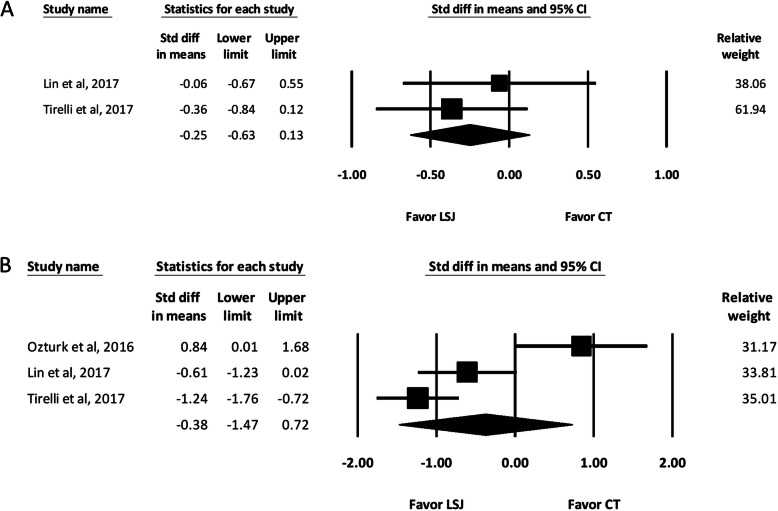
**a** Forest Plot of the Length of Hospital Stay. **b** Drainage Amount. Std diff in means, Standardized difference in means; CI, confidence interval; LSJ, LigaSure Small Jaw; CT, conventional technique

Two of the included studies reported the length of hospital stay [2, 6]. The pooled analysis showed no significant difference between the LSJ and conventional technique groups regarding the length of hospital stay (SMD, − 0.25; 95% CI, − 0.63 to 0.13) (Fig. 5).

#### Drainage amount

Three of the included studies reported the drainage amount [2, 3, 6]. The pooled analysis showed no significant difference between the LSJ and conventional technique groups regarding the drainage amount (SMD, − 0.38; 95% CI, − 1.47 to 0.72) (Fig. 5b).

### Publication bias

The funnel plots and the results of the Egger’s and heterogeneity tests are presented in eTable 4 in the Supplement. Egger’s test was positive for drainage amount (*P* = 0.031), indicating that the result may have been influenced by publication bias. The results of the Egger’s tests for other parameters indicated no apparent publication bias.

## Discussion

The present meta-analysis was conducted to evaluate the differences between LSJ and conventional techniques in ND in the existing English literature. According to our meta-analysis, the LSJ group demonstrated a significantly shorter operative time than the conventional group. In addition, the incidences of postoperative hematoma and surgical site infection were comparable between the two groups. The amount of intraoperative blood loss and the length of hospital stay also showed no significant intergroup differences. To our knowledge, this is the first time these two techniques in ND have been systemically reviewed and compared.

ND plays an essential role in surgical treatments for various head and neck cancers [1]. The extent of ND levels depends on the severity of head and neck cancer and subsequent treatment planning [17]. The conventional technique for ND relies on knot tying, surgical clips, cold instruments and electrocautery. However, several energy-based devices have been developed and used in recent years^7,9,10^. Developed in 1998, the LigaSure vessel sealing system utilizes bipolar radiofrequency to perform coagulation, and a feedback-controlled system automatically shuts off when the sealing process is completed [18, 19]. In the reported literature, several types of LigaSure devices have been used in head and neck surgeries. When using the older generation of the LigaSure vessel sealing system, Metzenbaum scissors are still required to cut the tissue after coagulation [20]. However, the LSJ device, which was introduced in 2010, was able to transect the tissue with a cutting blade immediately after vessel sealing and was expected to further facilitate surgical procedures [10, 21–23]. In the present meta-analysis, only the studies that compared LSJ with conventional ND were included to prevent possible bias from different generations of LigaSure vessel sealing instruments. The pooled results from our analysis revealed a significant reduction in total operative time in the LSJ group. The use of LSJ in head and neck surgeries, such as tonsillectomy, parotidectomy and thyroidectomy, has also been previously reported to speed up the surgical process. Our result was consistent with the studies above and confirmed the feasibility and efficacy of LSJ in ND. One previous study on thyroidectomy defined that a decrease of at least 10 min in operation duration was considered clinically relevant [24]. The pooled results from our analysis indicated statistical as well as clinical significance for surgical time reduction when LSJ is used in ND.

LigaSure devices have been used to facilitate and secure hemostasis in various open and endoscopic procedures. With the use of LigaSure, the risk of bleeding does not increase, and some studies have even shown reduced intraoperative blood loss in hemorrhoidectomy and laparoscopic gynecologic surgeries. The pooled result from our study indicated a reduction in intraoperative blood loss of approximately 14 ml in the LSJ group. However, this reduction did not reach statistical significance. Possible explanations may be that intraoperative blood loss during ND was recorded in only two studies, and additional data are needed for a more comprehensive analysis. In addition, LSJ was used mainly to speed up the vessel ligation procedure, and the dissection steps did not differ substantially from those of conventional ND. Other factors, such as the surgeon’s experience or the underlying condition of the patients, may also be potentially related to the amount of blood loss during ND [25].

Incidences of postoperative complications were analyzed in our study. The pooled results demonstrated that the incidences of postoperative hematoma and postoperative surgical site infection were comparable between the two groups. A previous study comparing LSJ and conventional thyroidectomy also indicated that the complication rate was similar between the two techniques [26]. During ND, careful dissection and ligature of vessels are reported to be extremely important steps. The development of the LSJ helps to simplify vessel ligation while achieving reliable hemostasis. However, careful dissection remains fundamental in ND. Several factors in addition to surgical instruments can also play contributing roles in the development of postoperative complications. The level of ND, baseline nutrition status, and underlying disease of patients are related to complications after ND in the literature [27–29].

The length of hospital stay, including both “medically necessary” and “discharge delay” periods, was similarly a multifactorial result [30]. The pooled results revealed that the length of hospital stay was not significantly different between the two groups. Our result also indicated that LSJ-assisted ND did not reduce the total amount of drainage. The drainage volume after ND has been reported to be increased in patients with older ager, antithrombotic treatment, and greater extent of surgery [31]. The purchase cost of LSJ is greater than conventional instruments; however, several authors believed that the purchase cost would be offset by reducing time-related charges for the surgical team and operating theatre [4, 6]. Conflicting results have also been reported, and more studies may be needed to elucidate the cost-effectiveness issue [32].

The authors acknowledge the limitations of the present study. First, only four studies were included in this meta-analysis, and more studies are required to confirm these results. Second, although one of the included articles was a randomized trial, other studies were also included given the lack of available data in the literature. Third, the results of this meta-analysis should be interpreted with caution given the potential publication bias and heterogeneity between the included studies. Despite these limitations, our meta-analysis still provides evidence for the use of different techniques in ND.

## Conclusions

In conclusion, compared with conventional techniques, LSJ-assisted ND significantly reduces the operative time. The main advantage of LSJ is to simplify the vessel ligation procedure and eliminate the need for knot tying and clips while securing hemostasis. The amount of intraoperative blood loss, the amount of postoperative drainage and the incidences of postoperative hematoma and surgical site infection were comparable between the two groups. In addition, the use of LSJ did not increase the length of hospital stay. Surgeons may consider using LSJ according to personal experiences, preferences, and cost-effectiveness criteria.

## Supplementary Information

**Additional file 1.**

## Data Availability

The datasets used and/or analyzed during the current study are available from the corresponding author on reasonable request.

## Abbreviations

LSJ: Ligasure small jaw
ND: Neck dissection
SMDs: Standardized mean differences
MDs: Mean differences
RDs: Risk differences

## References

[1] Robbins KT, Clayman G, Levine PA (2002). Neck dissection classification update: revisions proposed by the American head and neck society and the American Academy of Otolaryngology-Head and Neck Surgery. Arch Otolaryngol Head Neck Surg.

[2] Lin WJ, Wang CC, Jiang RS, Huang YC, Ho HCLS (2017). A prospective randomised trial of LigaSure small jaw® versus conventional neck dissection in head and neck cancer patients. Clin Otolaryngol.

[3] Ozturk K, Kaya I, Turhal G, Ozturk A, Gursan G, Akyildiz S (2016). A comparison of electrothermal bipolar vessel sealing system and electrocautery in selective neck dissection. Eur Arch Otorhinolaryngol.

[4] Suzuki K, Shimizu M, Sakagami T (2018). A comparison of short-term outcomes of neck dissection for head and neck cancers using Thunderbeat™, LigaSure™ or treatment without an energy-based device: A case controlled study. Int J Surg.

[5] Mishra N, Samal D, Kar IB (2018). Bipolar vessel sealing system versus suture ligation in selective neck dissection. J Maxillofac Oral Surg.

[6] Tirelli G, Del Piero GC, Valentinuz G (2018). New generation cut-and-seal devices in oral and oropharyngeal cancer resection: clinical and cost-effectiveness study. J Laryngol Otol.

[7] Gillespie MB, Stachiw ND, Way J, Lentsch EJ, Richardson MS, Nguyen SA, Day TA, Hornig JD (2010). Neural outcomes after plasma knife dissection: a pathologic study and clinical correlation. Head Neck.

[8] Pons Y, Gauthier J (2009). 2009; U-PE. Comparison of LigaSure vessel sealing system, harmonic scalpel, and conventional hemostasis in total thyroidectomy. Otolaryngol Head Neck Surg..

[9] Fakhry N, Michel J, Santini L, Lagier A, Turner F, Dessi P, Giovanni A (2012). Utility of the LigaSure vessel sealing system during major head and neck cancer surgery. J Laryngol Otol.

[10] Kanno C, Masubuchi T, Tada Y, Fushimi C, Matsuki T, Takahashi H, Okada T, Inomata T, Sasaki M, Niwa K, Machida T, Miura K (2018). Efficacy and safety of a vessel sealing system in oral cancer resection and reconstructive surgery. Acta Otolaryngol.

[11] Molnar C, Voidazan S, Rad CC, Neagoe VI, Roşca C, Barna L, Copotoiu C (2014). Total thyroidectomy with LigaSure small jaw versus conventional thyroidectomy - a clinical study. Chirurgia (Bucur).

[12] Khanna R, Khanna S, Bhadani S, Singh S, Khanna AK (2010). Comparison of Ligasure Hemorrhoidectomy with conventional Ferguson's Hemorrhoidectomy. Indian J Surg.

[13] Manouras A, Markogiannakis H, Genetzakis M, Filippakis GM, Lagoudianakis EE, Kafiri G, Filis K, Zografos GC (2008). Modified radical mastectomy with axillary dissection using the electrothermal bipolar vessel sealing system. Arch Surg.

[14] Higgins JP, Altman DG, Gøtzsche PC, Jüni P, Moher D, Oxman AD (2011). The Cochrane Collaboration's tool for assessing risk of bias in randomised trials. Bmj.

[15] Peterson J, Welch V, Losos M, Tugwell P (2011). The Newcastle-Ottawa scale (NOS) for assessing the quality of nonrandomised studies in meta-analyses.

[16] Davey J, Turner RM, Clarke MJ, Higgins JP (2011). Characteristics of meta-analyses and their component studies in the Cochrane database of systematic reviews: a cross-sectional, descriptive analysis. BMC Med Res Methodol.

[17] Ferlito A, Rinaldo A, Silver CE (2006). Neck dissection: then and now. Auris Nasus Larynx.

[18] Kennedy JS, Stranahan PL, Taylor KD, Chandler JG (1998). High-burst-strength, feedback-controlled bipolar vessel sealing. Surg Endosc.

[19] Kennedy J, Buysse S, Chandler J, Eggleston J, Taylor K, Thomsen S. Controlled radio frequency vessel sealing system for surgical applications. In: Proc. SPIE 3249, Surgical Applications of Energy; 1998. 10.1117/12.304336.

[20] Prokopakis EP, Lachanas VA, Karatzanis AD, Benakis AA, Velegrakis GA (2005). How we do it: application of Ligasure vessel sealing system in patients undergoing total laryngectomy and radical neck dissection. Clin Otolaryngol.

[21] Hwang SO, Jung JH, Park HY, Kim WW (2014). A prospective, randomized study between the small jaw® and the harmonic focus® in open thyroidectomy. Otolaryngol Head Neck Surg.

[22] Hirunwiwatkul P, Tungkavivachagul S (2013). A multicenter, randomized, controlled clinical trial of LigaSure small jaw vessel sealing system versus conventional technique in thyroidectomy. Eur Arch Otorhinolaryngol.

[23] Hahn CH, Sørensen CH (2013). LigaSure small jaws versus cold knife dissection in superficial parotidectomy. Eur Arch Otorhinolaryngol.

[24] Van Slycke S, Gillardin JP, Van Den Heede K, Minguet J, Vermeersch H, Brusselaers N (2016). Comparison of the harmonic focus and the thunderbeat for open thyroidectomy. Langenbeck's Arch Surg.

[25] BuSaba NY, Schaumberg DA (2007). Predictors of prolonged length of stay after major elective head and neck surgery. Laryngoscope.

[26] Yao HS, Wang Q, Wang WJ, Ruan CP (2009). Prospective clinical trials of thyroidectomy with LigaSure vs conventional vessel ligation: a systematic review and meta-analysis. Arch Surg.

[27] Dedivitis RA, Guimarães AV, Pfuetzenreiter EG, Castro MA (2011). Neck dissection complications. Braz J Otorhinolaryngol.

[28] Kerawala CJ, Heliotos M (2009). Prevention of complications in neck dissection. Head Neck Oncol.

[29] Park SY, Kim MS, Eom JS, Lee JS, Rho YS (2016). Risk factors and etiology of surgical site infection after radical neck dissection in patients with head and neck cancer. Korean J Intern Med.

[30] Lang DM, Danan D, Sawhney R, Silver NL, Varadarajan VV, Balamohan S, Bernard SH, Boyce BJ, Dziegielewski PT (2019). Discharge delay in head and neck free flap surgery: risk factors and strategies to minimize hospital days. Otolaryngol Head Neck Surg..

[31] Saito I, Hasegawa T, Iwata E, Yonezawa N, Arimoto S, Takeda D, et al. Postoperative drainage in head and neck surgery for oral cancer. Journal of Oral and Maxillofacial Surgery, Medicine, and Pathology. 2017;29(3):217–21. 10.1016/j.ajoms.2016.12.010.

[32] da Silva FB, Limoeiro AC, Del Bianco J, Teich V, Teich N, Teixeira JC (2012). Impact of the use of vessel sealing or harmonic scalpel on intra-hospital outcomes and the cost of thyroidectomy procedures. Einstein (Sao Paulo).

