# Supplementary material for: LigaSure small jaw versus conventional neck dissection: a systematic review and meta-analysis
Source: J Otolaryngol Head Neck Surg. 2021 Mar 29;50:21. doi: 10.1186/s40463-021-00504-2 (PMC8008542; doi:10.1186/s40463-021-00504-2)
Supplement: Supplementary file 1 — Additional file 1. [file 40463_2021_504_MOESM1_ESM.doc]

**eTable 1.** Literature Searches and Keywords

| - Medline   ("neck dissection" OR "cervical lymphadenectomy" OR "neck lymphadenectomy" OR "cervical lymph node dissection" OR "neck lymph node dissection") AND ("vessel sealing" OR "ligasure" OR "energy") |
| --- |
| - EMBASE   ('neck dissection'/exp OR 'neck dissection' OR 'cervical lymphadenectomy' OR 'neck lymphadenectomy' OR 'cervical lymph node dissection' OR 'neck lymph node dissection') AND ('vessel sealing' OR 'ligasure'/exp OR 'ligasure' OR 'energy'/exp OR 'energy') |
| - Cochrane library   (("neck dissection" OR "cervical lymphadenectomy" OR "neck lymphadenectomy" OR "cervical lymph node dissection" OR "neck lymph node dissection") AND ("vessel sealing" OR "ligasure" OR "energy")):ti,ab,kw |

eTable 2: Newcastle-Ottawa Scale Quality Assessment of Included Non-randomized Studies

| **Study ID** | **Selection** | | | | **Comparability*** | **Outcome** | | | **Total (**⋆**)** |
| --- | --- | --- | --- | --- | --- | --- | --- | --- | --- |
|  | Representativeness of the Exposed Cohort | Selection of the Non-Exposed Cohort | Ascertainment of Exposure | Demonstration that Outcome of Interest was not Present at Start of Study | Comparability of Cohorts on the Basis of the Design or Analysis | Assessment of Outcome | Follow-up was  Long Enough  for Outcomes  to Occur | Adequacy of  Follow-up of  Cohorts |  |
| Tirelli 2017 | ⋆ | ⋆ | ⋆ | ⋆ | ⋆⋆ | ⋆ | ⋆ | ⋆ | 9 |
| Suzuki 2018 | ⋆ | ⋆ | ⋆ | ⋆ | ⋆⋆ | ⋆ | ⋆ | ⋆ | 9 |
| Ozturk 2016 | ⋆ | ⋆ | ⋆ | ⋆ |  | ⋆ |  |  | 5 |

eTable 3: Risk of Bias Assessment of Included Randomized Studies

| **Study ID** | Random sequence generation | Allocation concealment | Blinding of participants and personnel | Blinding of outcome assessment | Selective reporting | Other biases |
| --- | --- | --- | --- | --- | --- | --- |
| Lin 2017 | Low | Unclear | High | Low | Unclear | Unclear |

**eTable 4:** Funnel plots

| **Parameter** | ***I^2^*** | **Egger’s Test** | **Funnel Plot** |
| --- | --- | --- | --- |
| Operative Time  (overall study group) | 81.725 | 0.717 | 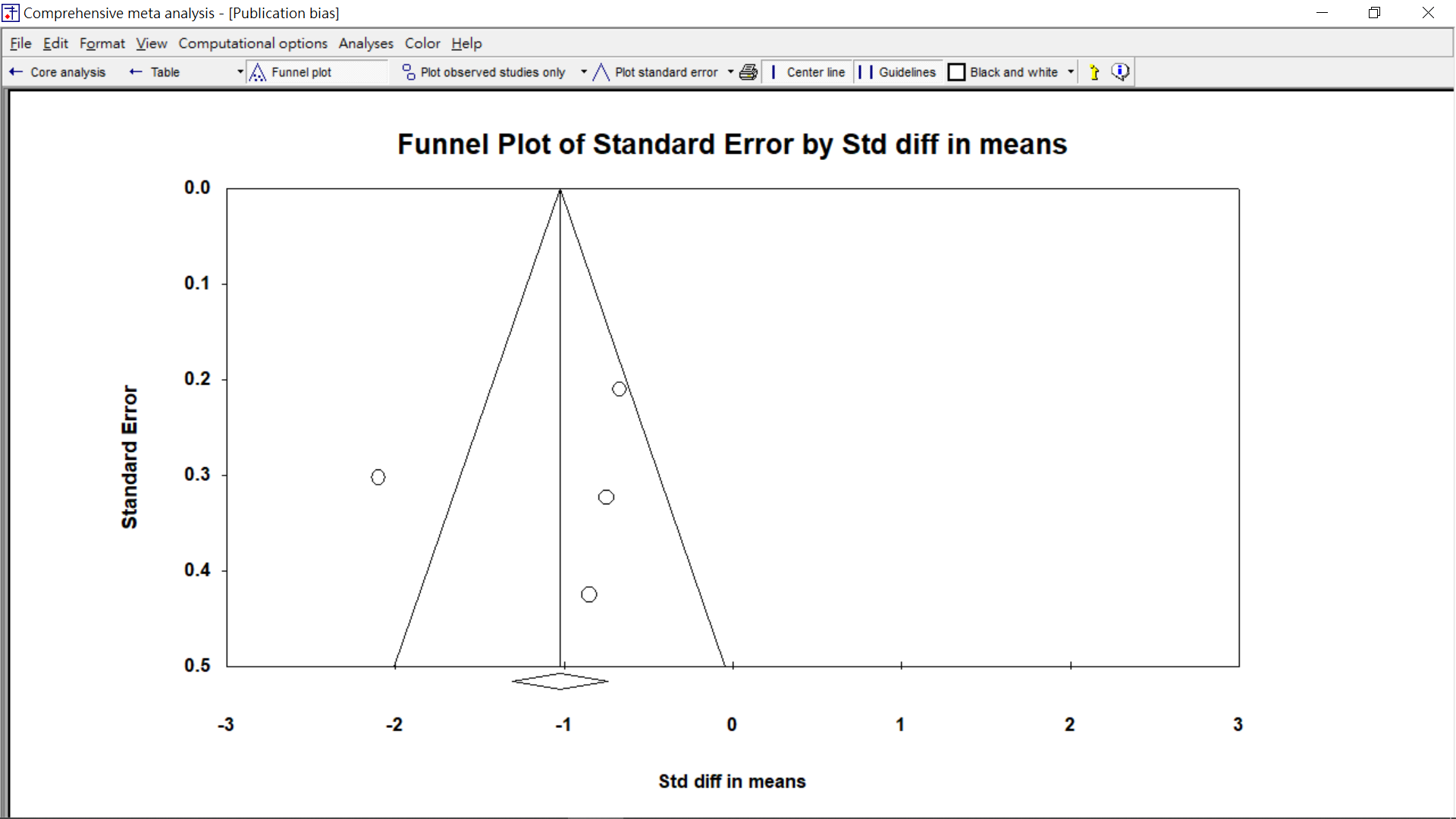 |
| Operative Time  (studies including only selective neck dissection) | 0.000 | X | X |
| Intraoperative blood loss | 0.000 | X | X |
| Postoperative hematoma | 0.000 | 0.833 | 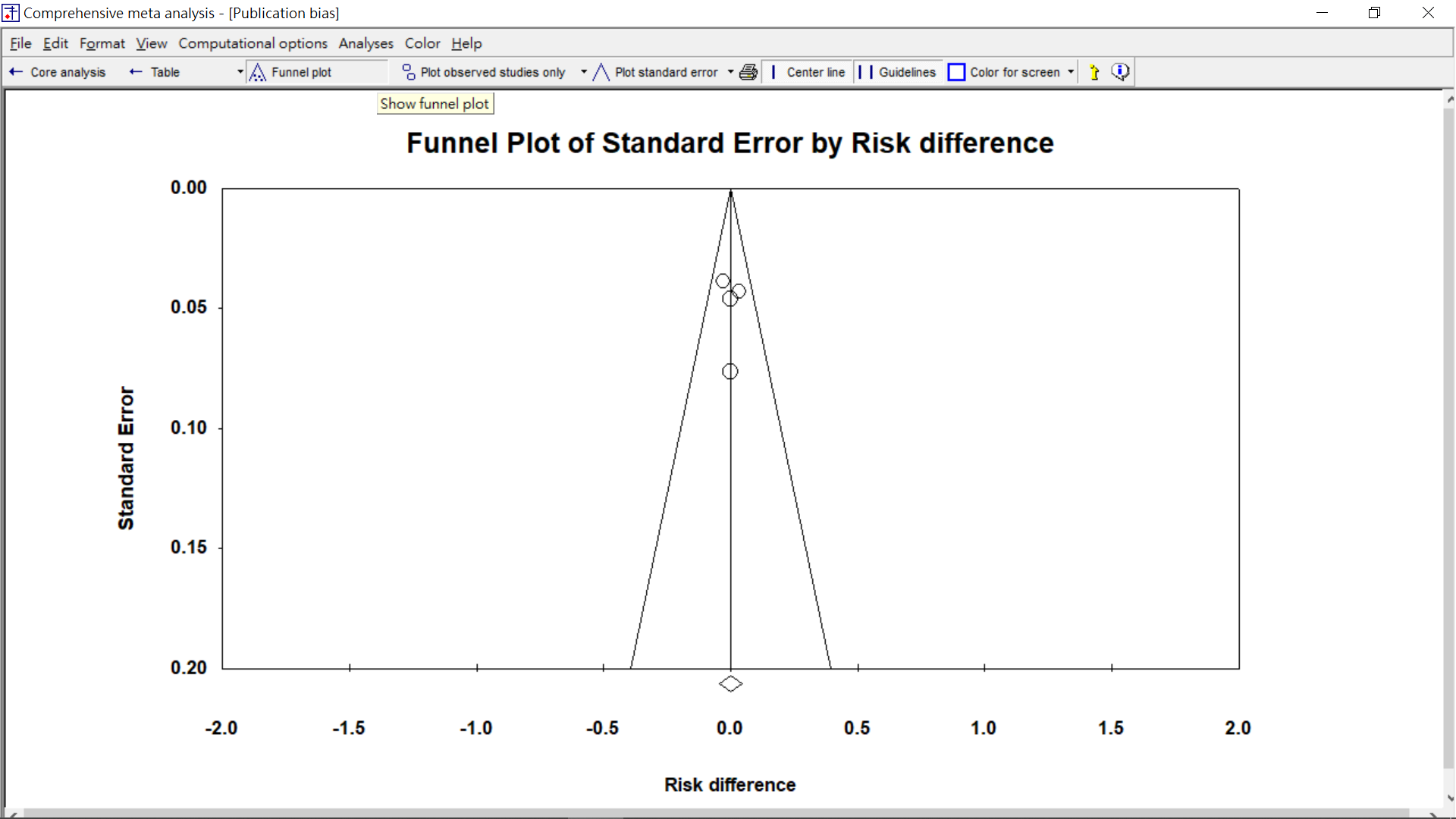 |
| Postoperative surgical site infection | 0.000 | 0.862 | 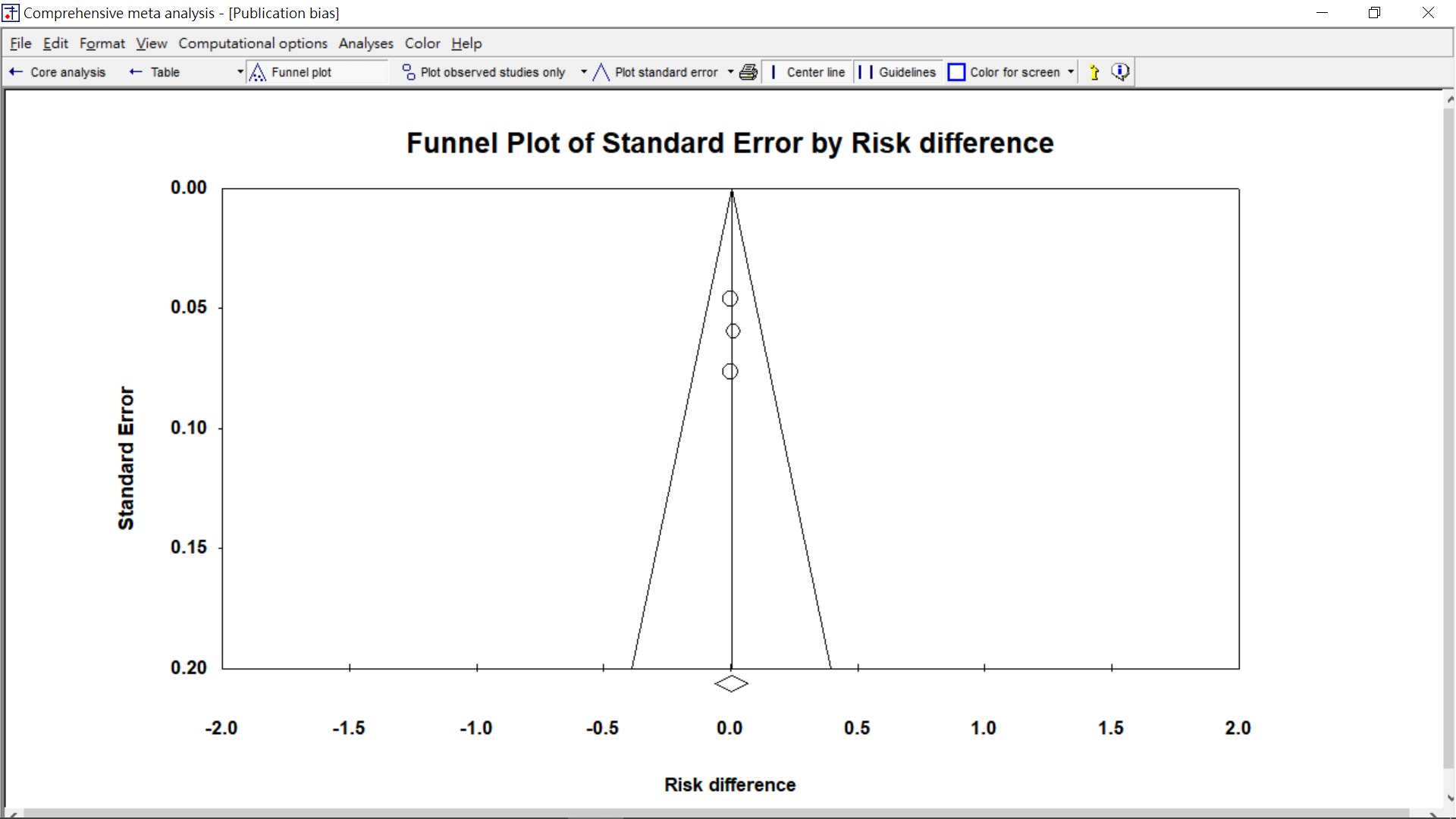 |
| Length of hospital stay | 0.000 | X | X |
| Drainage amount | 88.411 | 0.031 | 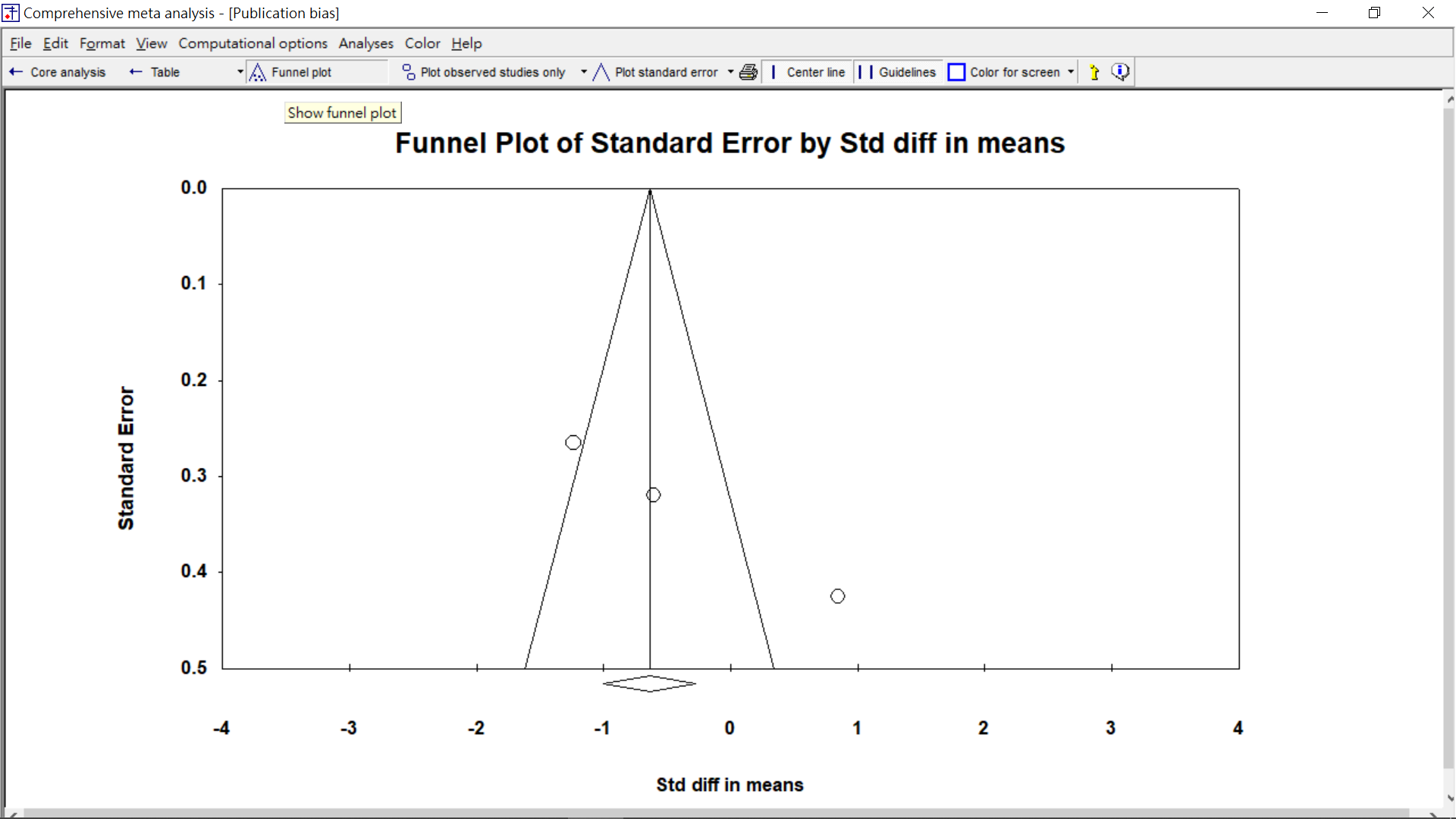 |
